# Clinical applications of resting coronary flow detection via transthoracic echocardiography

**DOI:** 10.3389/fcvm.2025.1641896

**Published:** 2025-11-06

**Authors:** Zixuan He, Ruohan Zhao, Xin Zhang, Liqiong Shi, Siyi Zhang, Jia Xu, Yinting Xiong, Qing Lv

**Affiliations:** Department of Ultrasound, Union Hospital, Tongji Medical College, Huazhong University of Science and Technology, Wuhan, China

**Keywords:** transthoracic echocardiography, resting, coronary flow, cardiovascular disease, clinical application

## Abstract

Beyond being a component of coronary flow velocity reserve, resting coronary blood flow is recognized as a clinically relevant measure, providing hemodynamic, diagnostic, and prognostic information across some cardiovascular diseases. With advancements in high-frequency transducers and imaging protocols, transthoracic coronary artery imaging has become increasingly non-invasive, practical, and useful. This review aims to summarize the clinical value and applications of transthoracic echocardiography (TTE) to assess coronary blood flow at rest.

## Introduction

Coronary flow velocity reserve (CFVR) was the original cornerstone of coronary hemodynamic assessment and remains a critical marker for major adverse cardiac events (MACEs). However, previous studies have demonstrated that high resting coronary flow velocity correlates with reduced CFVR (1). Other quantitative parameters derived from resting coronary flow, such as diastolic-to-systolic velocity ratio, the mosaic flow, and deceleration time, have demonstrated predictive value for coronary artery diseases or disease-specific hemodynamic alterations. Transthoracic echocardiography (TTE) has revolutionized coronary artery visualization through advancements in ultrasound imaging technology. Earlier studies have confirmed the feasibility of two-dimensional TTE for visualizing proximal coronary arteries (2, 3). Later studies have demonstrated the left anterior descending (LAD) artery flow by non-invasive two-dimensional and Doppler echocardiography, thereby opening a new field (4). The detection and application of coronary flow have since increasingly expanded to include the right coronary artery (RCA) and left circumflex artery (LCX), with a relatively lower detection rate and limited clinical emphasis (5, 6). Intramyocardial arteries have gradually garnered attention as potential markers of regional perfusion, complementing the global perfusion insights provided by epicardial coronary visualization. Resting flow assessment is non-invasive, cost-effective, and non-stressful compared with CFVR measurement. This review highlights the clinical application of underappreciated resting coronary flow parameters as feasible alternatives.

## TTE coronary detection

### Machine settings

A high-resolution, high-sensitivity phased-array ultrasound system is employed for coronary flow analysis. To ensure optimal blood flow visualization, pre-scanning adjustments are recommended. A high-frequency transducer, adult multifrequency, or pediatric probe is used according to the depth of the segment of interest (6). The velocity scale is set to an initial optimal Nyquist limit of 20–30 cm/s, and the wall motion filters are turned off, following a clinical consensus (7). The following supplementary settings are utilized while imaging with pulse Doppler: a 3–5 mm sample volume, compression set at 6, and no signal rejection. The Nyquist limit can be adjusted according to the optimal imaging requirements.

### Methods for coronary flow detection

Patients are examined in the left lateral position using a modified left parasternal acoustic window. Starting with the short-axis view of the aortic valve, the proximal segments of the LAD, LCX, and left main artery are visualized. The LAD artery courses along the anterior interventricular sulcus. To identify the mid-distal LAD artery, a long-axis view of the left ventricle is obtained. The mid-distal LAD artery is recognized as a tubular structure located within the anterior interventricular sulcus near the apex. The ultrasound beam is then tilted laterally and superiorly to delineate the anterior interventricular sulcus and locate the LAD artery under color Doppler monitoring, with flow direction from the base to the apex. The angle between the Doppler beam and the longitudinal axis of the coronary artery is minimized to optimize pulsed Doppler sampling. The sample volume is positioned within the vessel lumen for as much of the cardiac cycle as possible. Both the Doppler waveform and two-dimensional echocardiographic images are recorded (8). Additionally, modified apical four-, three-, and two-chamber views can be utilized to confirm the anatomic position of the LAD coronary artery.

With regard to the RCA, the proximal part is visualized in the short-axis view of the aortic valve. A shortened apical two- or three-chamber view makes it possible to display the distal branch in the posterior interventricular sulcus and posterior descending artery (PDA). According to practice guidelines, the modified apical four-chamber view, achieved by rotating the ultrasound probe clockwise by 50°–80° from the standard apical four-chamber position, allows identification of distal LCX flow in the left ventricular lateral wall (9).

In a study by Fusejima (4), the LAD artery Doppler flow signals were initially identified in 35% of healthy subjects and 50% of patients with various cardiovascular diseases. Fusejima reported a biphasic flow pattern and a higher velocity during diastole. The visualization of three major coronary arteries via Doppler echocardiography achieves a detection rate of 70%–80% without contrast enhancement (10, 11); however, this method is dependent on experience. A previous study has demonstrated that high-frequency imaging, harmonic imaging, and echo-contrast enhancement nearly achieve 100% visualization of LAD arterial segments (12). Due to practical limitations (depth and no clear anatomic marks) in visualizing the other two epicardial coronary arteries (RCA and LCX), most studies have focused on the LAD artery as the most accessible epicardial coronary artery (9). Despite the theoretical potential of evaluating all three main coronary arteries, technical challenges limit the routine assessment of these arteries. Thus, research priorities have focused on the LAD artery in epicardial coronary artery studies.

**Figure 1:**
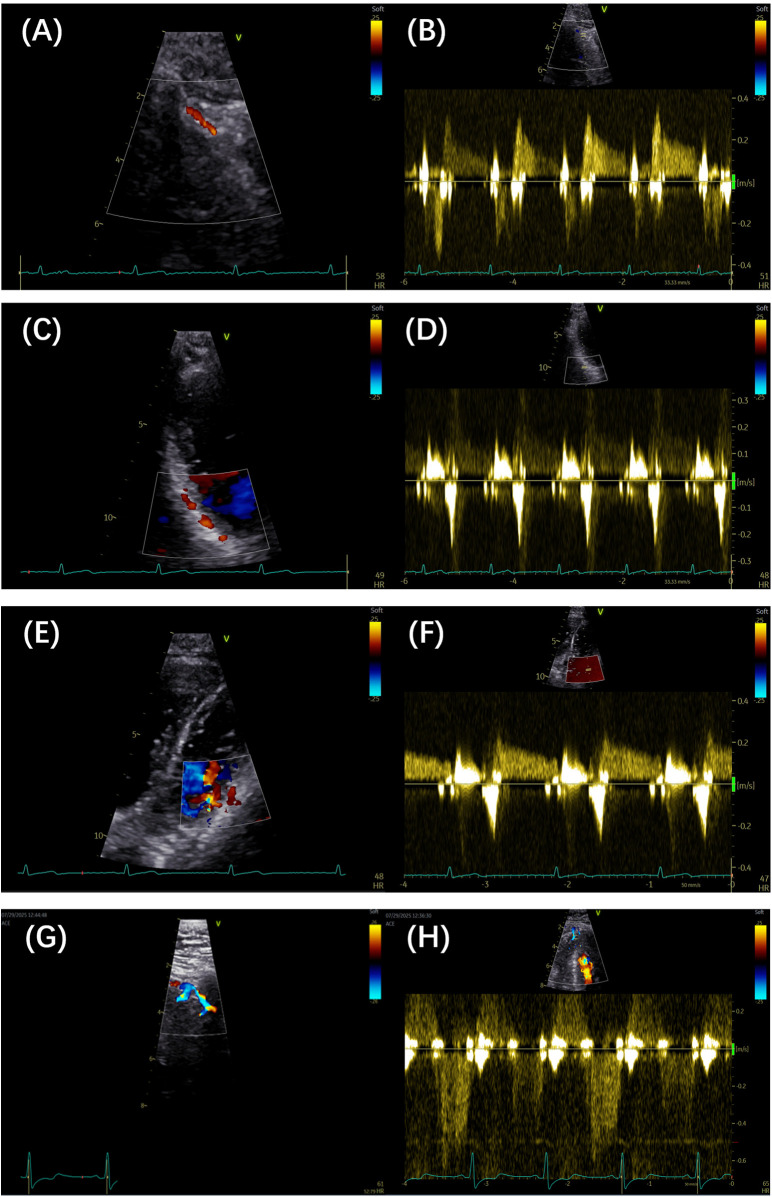
**(A,B)** Color Doppler and pulse Doppler detection of the LAD artery, respectively. **(C,D)** Color Doppler and pulse Doppler detection of the PDA, respectively. **(E,F)** Color Doppler and pulse Doppler detection of the LCX branch, respectively. **(G,H)** Color Doppler and pulse Doppler detection of septal branches, respectively. LAD, left anterior descending; PDA, posterior descending artery; LCX, left circumflex artery.

Advances in high-frequency TTE have demonstrated that intramyocardial perforators can be visualized and reflect myocardial perfusion (13). Sherrrid et al. (14) successfully recorded the septal perforator flow in patients with hypertrophic cardiomyopathy (HCM), patients with hypertensive left ventricular hypertrophy, and normal controls. To image the perforator arteries, short views between the apex and parasternal areas are used, complemented by modified two-chamber and parasternal views. Visualization of septal perforators in the mid- and apical septum is feasible using apical long-axis and off-axis views (14). To begin, left parasternal short-axis views at the level of the mitral valve annulus, chordae tendineae, and papillary muscles are used. Classical or modified two-, three-, or four-chamber views are then used to evaluate intramyocardial artery flow from the epicardium to the endocardium in the apical half of the left ventricular wall, applying color flow mapping with a low Nyquist limit. Table 1 summarizes the detection techniques, and Figure 1 illustrates clinical case examples.

**Table 1:** Detection techniques and views of main arteries.

Coronary arteries	Acquisition view	Detection position	Notices
Left anterior descending (LAD) artery	a) Short-axis view of the aortic valve b) Modified parasternal long-axis view c) Modified apical four-, three-, and two-chamber views	Left main coronary artery, proximal LAD artery, and mid-distal LAD artery (most used)	a) Diastolic-dominant flow with color Doppler monitoring b) Focused view of the anterior interventricular groove with avoidance of mistaken intracavitary flow c) The angle between the Doppler beam and the longitudinal axis of the coronary was as small as possible
Right coronary artery (RCA)	a) Short-axis view of the aortic valve b) Shortened apical two- or three-chamber view	Proximal RCA, mid-RCA, posterior descending coronary artery (PDA) (mostly a branch of the right coronary artery)	a) Diastolic-dominant flow with color Doppler monitoring b) Tricuspid annulus or right atrioventricular groove as anatomical landmarks of RCA; mostly difficult to detect the Doppler signal due to the perpendicular angle c) PDA walking in the posterior wall of the left ventricle; dynamically adjusted frequency due to distance d) The angle between the Doppler beam and the longitudinal axis of the coronary was as small as possible
Left circumflex coronary artery (LCX)	a) Short-axis view of the aortic valve b) Modified apical four-chamber view	Proximal LCX and distal LCX	a) Diastolic-dominant flow with color Doppler monitoring b) Focus on the lateral wall of the left ventricle; dynamically adjusted frequency due to distance c) The angle between the Doppler beam and the longitudinal axis of the coronary was as small as possible
Intramyocardial perforator	Short views between the apex and parasternal space	Branches of the LAD artery, LCX, and RCA	The angle between the Doppler beam and the longitudinal axis of the coronary was as small as possible

### Assessment of parameters

When coronary blood flow is interrogated, spectral Doppler is applied to obtain the coronary flow spectrum and extract its features. The onset of systole is defined by the peak of the R wave on the electrocardiogram, while the beginning of diastole is marked by the end of the T wave, thereby distinguishing systolic from diastolic flow in the absence of electrocardiographic abnormalities.

**Figure 2:**
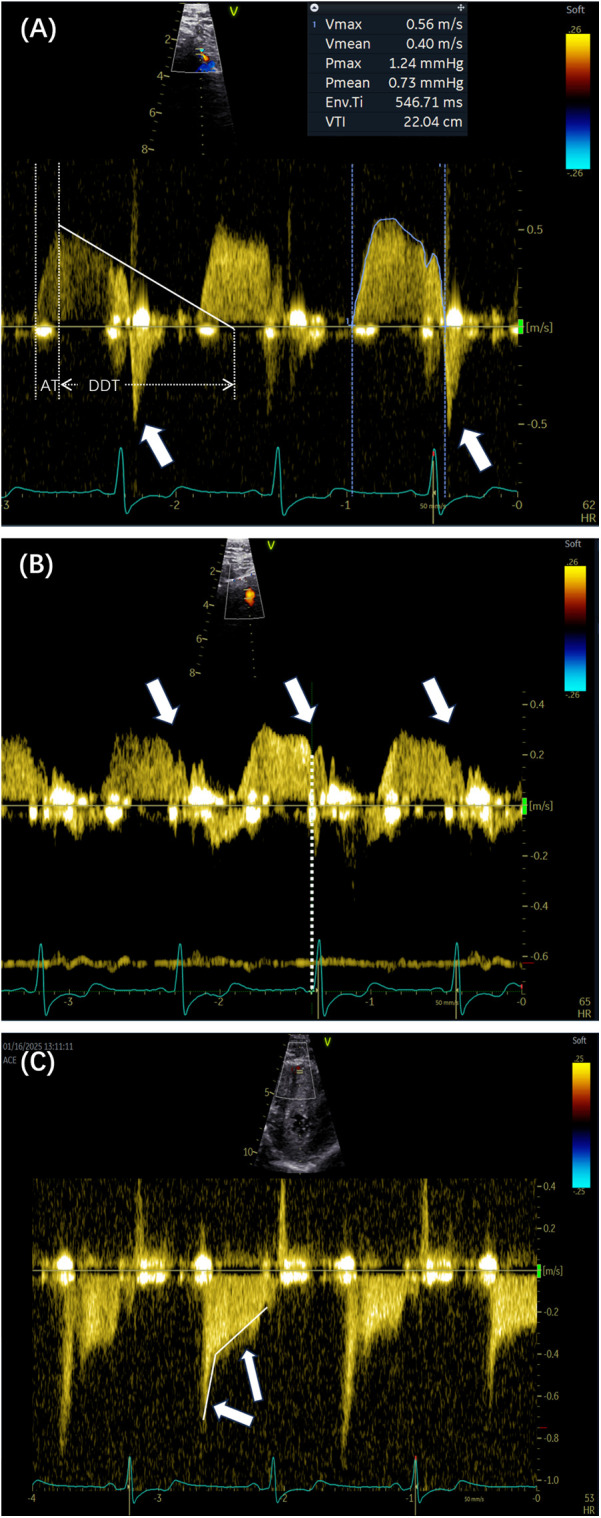
**(A)** The outline of the Doppler spectrum was traced to calculate various parameters, including the peak velocity, mean velocity, and velocity–time integral. The white lines indicate the DDT and AT. The arrows show the presence of SFR (arrows). **(B,C)** Two Doppler spectrum features are indicated by the sudden dip in end-diastolic velocity (arrows) and the corresponding time point of the dip (dashed lines). The square root sign patterns are indicated by arrows. DDT, diastolic deceleration time; AT, acceleration time; SFR, systolic flow reversal.

Peak flow velocity is defined as the highest instantaneous velocity within the systolic or diastolic phase, as measured from the coronary flow Doppler spectrum. The velocity is obtained by tracing the outer envelope of the Doppler signal using dedicated post-processing software, which automatically calculates the peak and mean velocities. The peak diastolic–systolic velocity ratio, peak stenotic to pre-stenotic diastolic velocity ratio, and pre-stenotic to stenotic mean diastolic flow velocity ratio are then calculated. The mosaic flow is regarded as a turbulent flow, characterized by locally persistent color aliasing due to rapid velocity exceeding the Nyquist limit. In detecting coronary stenosis, rescaling of the Nyquist limit of color flow Doppler at 0.48 m/s has been shown to be effective (15). Diastolic deceleration time (DDT) is measured from the peak diastolic velocity to the point where the initial decay slope line intersects with baseline. Diastolic pressure half-time (DPH) is the time interval required for peak diastolic velocity to decrease to $1/\sqrt{2}$ of its initial value. Acceleration time (AT) refers to the time from the onset of flow to the peak velocity on the Doppler velocity curve. Systolic flow reversal (SFR) is a reversal of blood flow direction during ventricular systole, in the opposite direction to diastolic forward flow. Two unique coronary flow Doppler patterns have been identified as follows: the end-diastolic velocity sudden dip, which is caused by atrial contraction, is defined as a transient decrease in coronary flow velocity occurring between the P wave and the peak of the R wave on the electrocardiogram; and a unique square root sign is characterized by a steep velocity slope and followed by a plateau phase. Figure 2 illustrates representative parameters of these patterns.

## Coronary flow velocity clinical applications by disease category

Given the essential role of CFVR in the prognostication of adverse outcomes and myocardial dysfunction (16–18), the resting coronary flow velocity has often been overlooked. The clinical value of coronary blood flow velocity is summarized below, focusing on coronary artery disease (CAD) and velocity alterations, emphasizing HCM.

### CAD

Patients with CAD exhibit coronary flow abnormalities, such as epicardial coronary stenosis, myocardial ischemia, myocardial infarction, and other conditions. An elevated coronary velocity indicates worsening coronary flow, a sign of arterial narrowing, or endothelial dysfunction. This observation is based on the principle that blood flow velocity is inversely proportional to the cross-sectional area of the vessel at a given level. Increased myocardial oxygen demand may contribute to other pathogenic mechanisms. Additionally, low velocity also suggests poor perfusion. To date, related studies have focused primarily on two main aspects—indicators of prognostication or MACEs and assessment of epicardial coronary stenosis (Table 2).

**Table 2:** Articles on resting coronary flow velocity in relation to CAD in chronological order.

Reference	Year	Cutoff	Analysis
Lauro et al. (19)	2024	Mid-distal LAD arterial flow velocity of 32 cm/s	High resting LAD arterial velocity was related to worse survival in preserved LVEF patients with chronic coronary syndromes
			A flow velocity of 32 cm/s was indicative of coronary flow velocity reserve
Zagatina et al. (5)	2023	Any proximal arteries with >64–67 cm/s; any coronary segments with a velocity of >65 cm/s	A maximal coronary flow velocity of 67 cm/s in the left main artery, proximal LAD artery, or proximal LCX was the best predictor for death. A velocity of 66 cm/s was the best predictor for death or myocardial infarction, and a velocity of 64 cm/s was a significant predictor of all MACEs
			Three groups classified by proximal segment velocity >65 cm/s, middle segment velocity >65 cm/s, and whole segment velocity <65 cm/s indicated high risk (5.6%), intermediate risk (2.3%), and low risk (0.3%) of death or myocardial infarction per year, respectively
Watanabe (6)	2017	Systolic LAD arterial average velocity of 6.5 cm/s	An average systolic velocity of 6.5 cm/s was the optimal cutoff value to predict viable myocardium
Lee et al. (21)	2003	Diastolic distal LAD arterial peak velocity of 25 cm/s	A distal diastolic LAD arterial peak velocity >25 cm/s distinguished TIMI-3 from TIMI-2 coronary reperfusion before emergency coronary intervention
Caiati et al. (24)	2023	Accelerated stenotic flow increment with values of 109% and 16%	An AsF% of 109% distinguished LAD arterial diameter narrowing greater than or less than 50%, while an AsF% of 16% identified narrowing but less than 50% from no coronary stenosis.
Gaibazzi et al. (29)	2019	Peak diastolic–systolic velocity ratio of 1.7	The reduced ratio was strongly related to significant coronary artery stenosis
Holte et al. (15)	2015	a) Peak stenotic (aliasing flow) to pre-stenotic velocity ratio of 2 b) Mosaic flow at Nyquist limit of 48 cm/s	A ratio of >2 and local mosaic flow no less than 48 cm/s were used to diagnose significant coronary disease in the left main coronary artery, LAD artery, LCX, and RCA
Vegsundvåg et al. (28)	2014	Peak diastolic septal perforating branch velocity of 57 cm/s	A velocity of >57 cm/s reflected occluded contralateral artery
Holte et al. (30)	2013	Post-stenotic peak diastolic-to-systolic velocity ratio of 1.68	The reduced ratio identified functionally significant coronary artery stenosis
Higashi et al. (27)	2013	Coronary velocity in color and pulse Doppler of 92 cm/s and 81 cm/s, respectively	A velocity greater than the optimal cutoffs in the proximal left coronaries indicated severe stenosis (diameter stenosis >70%) with high accuracy
Okamura et al. (20)	2005	A grading system with classified standards, including disappearance of systolic anterograde flow	Coronary flow abnormal patterns predicted microvascular dysfunction. When evaluating myocardium damage, the disappearance of anterograde flow was the last hemodynamic change considered
Daimon et al. (31)	2005	Diastolic-to-systolic flow peak velocity ratio of 1.6 and mean velocity ratio of 1.5	The ratios were used to physiologically estimate the absence or presence of reversible perfusion defects
Hozumi et al. (25)	2000	Pre-stenotic to stenotic (aliasing flow) mean diastolic flow velocity ratio of 0.45	The ratio of 0.45 was an optimal cutoff used to detect restenosis after coronary angioplasty
Krzanowski et al. (26)	2000	Stenosis to the adjacent segment peak velocity ratio of 2 or a local velocity of 2 m/s	A ratio of >2 or a local velocity of >2 m/s indicated the presence of stenosis with good specificity

LAD, left anterior descending; LCX, left circumflex coronary artery; MACEs, major adverse cardiac events; TIMI, thrombolysis in myocardial infarction; AsF, accelerated stenotic flow; RCA, right coronary artery; CAD, coronary artery disease.

### CAD: prognosis indicators

**Figure 3:**
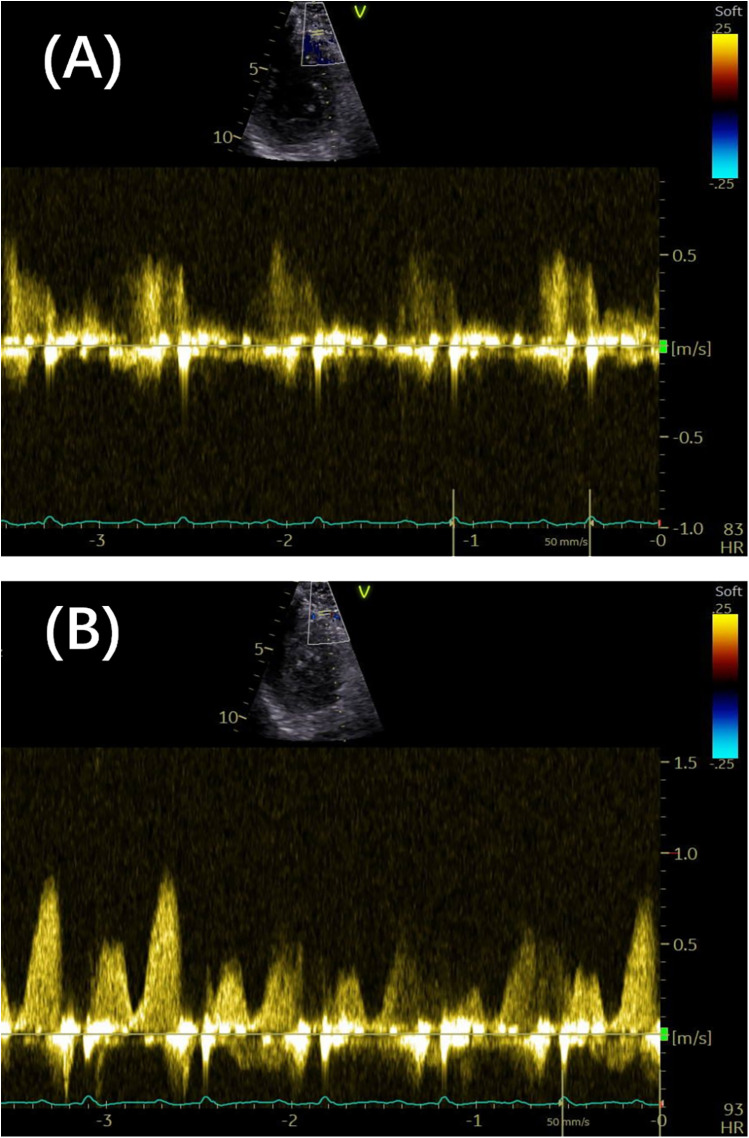
**(A)** The distal LAD arterial flow velocity at rest was 0.58 m/s. **(B)** The distal LAD arterial flow velocity under vasodilator-induced hyperemia was 0.88 m/s. The coronary flow velocity reserve was 1.58. LAD, left anterior descending.

Previous studies have indicated that resting coronary velocity is higher when CFVR is lower. Several retrospective studies have highlighted the prognostic significance of coronary velocity, with prospective observations gradually complementing these findings. In a prospective study involving 747 patients, Zagatina et al. (5) evaluated the proximal left-sided artery segment and middle segments, with a resting coronary velocity exceeding 64–67 cm/s serving as the cutoff for predicting death, myocardial infarction, and MACEs. Additionally, the value of 65 cm/s stratified the patients into the three following groups: high risk of death or myocardial infarction at the incidence of 5.6% per year, as classified by increased velocity in the proximal left-sided artery segments; moderate risk at 2.3% per year, as stratified by increased velocity in the middle segments; and low risk at 0.3% per year, as characterized by any segment maximal velocity below 65 cm/s. Subgroup analyses of patients with known CAD, suspected CAD, arterial hypertension, valvular disease, and chest pain with unknown reason also supported the positive correlation between higher coronary velocity and increased incidence of death, myocardial infarction, and MACEs (5). A resting coronary flow velocity cutoff of 32 cm/s has also been associated with worse outcomes in patients with chronic coronary syndromes and preserved left ventricular ejection fraction (LVEF), adding incremental prognostic value to CFVR assessments (19). The emphasis on resting flow velocity is justified, as it should not merely serve as an auxiliary parameter for CFVR measurement. Increased baseline velocity may mechanistically contribute to reduced CFVR. Figure 3 illustrates high resting velocity coexisting with reduced CFVR.

Reduced or absent systolic forward flow velocity exhibits predictive value. An average systolic velocity, with an optimal cutoff value of 6.5 cm/s, can predict viable myocardium (6). Absent systolic flow has been used to additionally estimate the severity of microvascular dysfunction and predict impairment in left ventricular function (20).

Low Doppler flow values represent a low perfusion state. It has been reported that a thrombolysis in myocardial infarction (TIMI) risk score—a semiquantitative index of myocardial tissue perfusion—of ≤2 in acute myocardial infarction (AMI) patients is related to adverse events. A diastolic peak distal LAD arterial flow velocity of 25 cm/s has demonstrated high diagnostic efficacy for distinguishing a TIMI risk score of 3 from a TIMI risk score of ≤2 prior to coronary intervention, with 77% sensitivity and 94% specificity (6, 21). A TIMI risk score of 3 exhibits a wide range of velocity and partially overlaps with that of a TIMI risk score of 2, resulting in low sensitivity. These findings align with previous studies demonstrating increased velocity with a TIMI risk score of 3 and reduced velocity with TIMI risk scores ranging from 0–2 according to intracoronary Doppler (22). Large clinical studies have shown better survival outcomes among patients who exhibit a normal or a TIMI risk score of 3 compared with those with incomplete reperfusion (TIMI ≤ 2). A coronary slow flow phenomenon is distinguished by a strong inverse correlation between coronary blood flow peak and mean velocities measured by TTE and TIMI frame count (23).

### CAD: stenosis assessment

The presence of diastolic accelerated stenotic flow (AsF) in the entire LAD artery indicates coronary stenosis, with different cutoffs of AsF increments across subgroups of stenosis explaining impaired CFVR (24). Baseline velocity elevations reliably identify atherosclerotic stenosis and microvascular disease only when systemic inflammation, hypertrophy, and anemia are excluded. Localized aliasing flow detected by color Doppler reflects artery stenosis. A peak stenotic to pre-stenotic velocity ratio exceeding 2.0 serves as a highly specific cutoff value for diagnosing significant coronary artery stenosis in the left main coronary and three main coronary arteries (15). A pre-stenotic to stenotic mean diastolic flow velocity ratio of <0.45 has been reported to effectively diagnose significant restenosis after percutaneous coronary intervention, with a sensitivity of 86% and a specificity of 93% (6, 25). A local maximal velocity exceeding 2 m/s has also been considered as stenosis (26). Mosaic flow, which is characterized by persistent color flow Doppler aliasing at a velocity scale of >48 cm/s, further supports the presence of significant stenosis (15). Additionally, invasive techniques provide complementary diagnostic value, particularly in cases with severe calcification and in-stent stenosis, where coronary computed tomography angiography may fail. Higashi et al. reported that optimal values for color Doppler and pulse Doppler to diagnose severe proximal left coronary artery stenosis are 92 cm/s (sensitivity of 100% and specificity of 90%) and 81 cm/s (sensitivity of 100% and specificity of 85%), respectively (27). Coronary artery occlusion may coexist with intramyocardial and epicardial collateral formation. Accelerated anterograde flow velocities exceeding 57 cm/s in septal perforator branches indicate collateral supply to contralateral coronary occlusion (28). A spike-like flow with high velocity in the early systolic to apex has been observed in patients with coronary artery bypass grafting; this flow is associated with passing the stenotic segment, as confirmed by coronary angiography (4).

The peak diastolic–systolic velocity ratio of the LAD artery at rest serves as a strong predictive marker for obstructive CAD (stenosis >50%) (29, 30). A peak diastolic–systolic velocity ratio of <1.7 outperforms other parameters, such as a coronary flow reserve of <2 [odds ratio (OR) 11.18 vs. 2.88], as well as clinical variables and wall motion abnormalities (OR 11.18 vs. 1.53) (29). In addition, the mean diastolic–systolic velocity ratio with a cutoff of 1.5 demonstrates equivalent utility in estimating the severity of LAD arterial stenosis, with a sensitivity of 81.8% and a specificity of 85.7% (31).

### HCM

Hemodynamic abnormalities in HCM arise from increased oxygen demand and concomitant insufficient capillary density. However, the velocity abnormality values need further exploration and validation. To date, the clinical values of coronary flow velocity in HCM remain insufficiently defined, and the current findings are summarized mainly in a descriptive context.

Compared to healthy controls, patients diagnosed with HCM exhibit a significantly reduced systolic peak blood velocity and systolic velocity–time integral (8), with no significant differences in these parameters between subgroups categorized by symptoms or left ventricular outflow tract obstruction.

Higher diastolic phase velocities in the LAD artery have been observed in HCM (32), and these higher velocities are attributed to an increase in oxygen demand. When patients with HCM are stratified into non-obstructive and obstructive HCM subgroups, diastolic flow velocity remains abnormally elevated (33, 34). Coronary flow velocity abnormalities are correlated with septal thickness rather than obstruction severity or pressure gradient (35). Thus, coronary flow recovery may be a marker to assess medication efficacy.

Compared with epicardial coronary arteries in HCM (primarily the LAD artery), intramyocardial small coronary arteries exhibit a significantly higher average diastolic peak flow velocity and velocity–time integral. In normal controls, no significant differences exist between the LAD artery and small coronary arteries (36). The diastolic peak velocity, mean diastolic velocity, and diastolic velocity–time integral in the septal perforator in HCM are higher than in hypertensive left ventricular hypertrophy, while there are no differences in these parameters between healthy subjects and individuals with hypertensive left ventricular hypertrophy. Epicardial coronary arteries can dilate and accommodate increased demand, normalizing velocity, whereas intramyocardial arteries fail to enlarge and remain significantly narrower (14).

### Other conditions

High coronary velocity serves as a long-term prognostic indicator, enabling identification of high-risk patients with MACEs among consecutive unselected cohorts referred for routine echocardiography examination due to chest pain, shortness of breath, and known cardiovascular diseases. In proximal and middle left-sided coronary segments (left main artery, LAD artery, and LCX), velocities exceeding 64–67 cm/s are associated with increased risks of mortality and MACEs (5). In proximal left-sided coronary segments, velocities exceeding 78 cm/s predict MACEs in the short term, irrespective of echocardiography results or other clinical data, and velocities exceeding 97 cm/s predict severe MACEs in the next 10.5 months (37). In unselected elderly patients, high coronary flow velocity (>81 cm/s) in the proximal segments of left-sided arteries is a significant predictor for MACEs (38). Many studies have demonstrated the value of high coronary velocities (39, 40).

Many studies have reported alterations in coronary blood flow velocity in patients with certain cardiovascular diseases, yet the clinical utility of these findings remains unexplored. These changes may represent a consequence of the underlying pathophysiological processes.

Aortic diseases affect coronary flow due to the perfusion pressure dependent on blood in the aortic sinuses. In patients with severe aortic insufficiency, the LAD arterial diastolic flow velocity is lower than the LAD arterial systolic flow velocity, and the diastolic flow terminates prematurely without lasting the entire diastolic phase. The LAD arterial diastolic flow velocity is higher in individuals with aortic stenosis than in healthy controls (4). In aortic stenosis patients, the peak and mean diastolic flow velocities in the LAD and intramyocardial coronary arteries are proportional to the mean aortic valve gradient (41). Reversed systolic flow normalizes after aortic valve replacement, despite a lack of change in the degree of hypertrophy, suggesting that ventricular hypertrophy caused by different etiologies may involve distinct pathophysiological mechanisms.

Increased baseline coronary flow velocity and decreased baseline microvascular resistance have been reported in short-duration patients with a history of diabetes mellitus of <10 years (42). High resting velocity is associated with insulin resistance in patients with type 2 diabetes, and this association has been attributed to abnormal metabolism (43). Coronary velocity changes imply impaired coronary microvascular function, thus explaining the mechanism for reduced CFVR.

Systolic and diastolic peak velocities are increased in patients with hypertrophy caused by hypertension (8). Pulse pressure, a parameter that reflects arterial stiffness and depends on conduit artery compliance, is associated with resting coronary flow velocity but is not significantly associated with CFVR (44). Higher pulse pressure, associated with impaired diastolic function, is correlated with higher coronary velocity and reduced CFVR, suggesting that increased baseline flow represents a compensatory mechanism.

## Coronary flow pattern clinical applications by disease category

Alterations in hemodynamics and myocardium structure affect microvascular and epicardial circulation, leading to coronary flow abnormalities. To the best of our knowledge, several parameters related to coronary flow patterns, such as DDT, DPH, bidirectional or retrograde systolic flow, and AT in the systolic phase, have been identified as clinically useful. The disease-related aspects, focusing mainly on CAD, are discussed below.

### CAD

CAD, characterized by coronary stenosis, has variable recovery outcomes. The coronary flow itself may serve as a predictive measure (Table 3). The association between DDT and cardiac function has been investigated. Watanabe et al. (45) reported that LVEF is significantly higher in patients with a longer DDT (>600 ms) compared with patients with a shorter DDT in both the acute and chronic phases of anterior AMI. Tani et al. (46) reported that a rapid DDT in the LAD and intramyocardial arteries identifies left ventricular remodeling and wall motion recovery after an anterior AMI. Some researchers have suggested that a DDT of <600 ms and SFR are markers of severe microvascular injury. Analyses using Doppler guidewire have confirmed that short diastolic DDT and SFR predict complications and in-hospital survival (47). A DDT of 600 ms is an optimal predictor for a viable myocardium (6). Saraste et al. (48) proposed that a DDT of <190 ms in the acute phase is strongly associated with a non-viable myocardium in reperfused AMI. Shintani et al. (49) reported that a DDT measured on Day 2 after reperfusion implies a functional outcome with coronary flow recovering toward normalization over time. A DDT >600 ms within 3 days after coronary angioplasty predicts a viable myocardium, with a sensitivity of 78% and specificity of 84%–92% (50). Persistent microvascular impairment in reperfused anterior AMI, defined as DDT of <600 ms on Day 7, is associated with left ventricular remodeling (49). Using Doppler guidewire and myocardial contrast TTE, previous studies have demonstrated that the diastolic deceleration slope is steeper in patients with AMI accompanied by the no-reflow phenomenon compared with those without no-reflow (51, 52). The no-reflow phenomenon is characterized by a steep diastolic deceleration slope with a DDT of <600 ms (53). Shortening of the LAD arterial DPH (<265 ms) indicates anterior permanent myocardial damage and scarred myocardial tissue, with a sensitivity of 79% and a specificity of 94% (54). The DDT and DPH are interdependently connected. Retrospective and prospective studies have reported that a short DPH predicts poor recovery in patients with anterior wall AMI after primary percutaneous transluminal coronary angioplasty or stenting (55). Receiver-operating characteristic analysis has indicated that a DPH of >300 ms is a useful cutoff. Using transthoracic Doppler echocardiography, Agostini et al. (56) identified distinct coronary velocity patterns in the LAD artery after primary percutaneous coronary intervention, and they reported that these patterns correlate with different contractile functions, clinical outcomes, and complication burdens. The deceleration shortens after AMI initiates ventricular remodeling and LVEF impairment, and it is the first flow change that is characterized, making it a sensitive and early indicator of capillary damage and reduced coronary capacity (20, 51). Tasaki et al. (57) reported that coronary flow characteristics reflect the artery patency after coronary artery bypass grafting through visualizing the existence of mosaic flow, retro flow, and sufficiently high anterograde flow. The DDT and deceleration slope reflect microvascular resistance conditions caused by cellular injury, local inflammation, interstitial edema, extravascular stress, stretching, and remodeling of the infarct region (58). Similar mechanisms underlie other flow characteristics, such as SFR and absent systolic anterograde flow.

**Table 3:** Articles on TTE-detected resting coronary flow patterns in relation to CAD in chronological order.

Reference	Year	Cutoff	Analysis
Giga et al. (53)	2023	DDT of infarct-related artery of 886 ms	A DDT value <886 ms indicated large, fixed perfusion abnormalities
Watanabe and Usui (45)	2021	a) Early SFR, defined as a reversal flow showing a peak velocity of 10 cm/s and duration >60 ms at early systole b) DDT of 600 s as a cutoff value	a) The presence of early SFR predicted impaired chronic cardiac function and a higher incidence of MACEs b) The DDT predicted chronic cardiac function
Vegsundvåg et al. (28)	2014	Retrograde flow in the main coronaries and intramyocardial branches	Collateral flow was accompanied by occluded coronaries
Takemoto et al. (62)	2014	Percentage acceleration time of 60%	Coronary stenosis was diagnosed with a 60% acceleration time even when accompanied by microvascular dysfunction
Boshchenko et al. (12)	2009	Flow reversal in epicardial collaterals, including the distal LAD artery, obtuse marginal branches, and PDA, as well as in intramyocardial collaterals, such as the LAD arterial septal branch and RCA septal branch	Coronary occlusion was diagnosed
Karatasakis et al. (54)	2008	Diastolic pressure half-time of 265 ms	The cutoff value of diastolic pressure half-time identified the presence or absence of a scarred anterior myocardium
Saraste et al. (48)	2007	DDT at 190 ms on Day 3 after AMI	The DDT was significantly longer in viable myocardium than in partially viable or non-viable myocardium. A DDT <190 ms predicted non-viable myocardium
Tani et al. (46)	2007	LAD arterial DDT value of 600 ms as a cutoff	A rapid DDT of the LAD artery and intramyocardial artery predicted a higher wall motion score index and left ventricular end-diastolic volume during the chronic phase after anterior AMI
Agostini et al. (56)	2006	Flow patterns: a) Antegrade systolic flow and slow diastolic deceleration slope b) Reduced or absent systolic flow and rapid diastolic deceleration slope c) Presystolic retrograde flow and rapid diastolic deceleration slope	Rapid diastolic deceleration resulted in more complications and left ventricular remodeling
Tasaki et al. (57)	2006	Flow characterized by mosaic flow at the anastomosis site, distal anterograde flow, and proximal retrograde flow	The presence of flow characterized the graft patency
Okamura et al. (20)	2005	A grading system classified according to short DDT (cutoff value of 600 ms), appearance of SFR, and disappearance of systolic anterograde flow	Coronary flow abnormal patterns, with the hemodynamic change order during the myocardium damage process (first to last) of short DDT, SFR, disappearing systolic anterograde flow, predicted microvascular dysfunction
Shintani et al. (49)	2004	DDT cutoff value of 600 ms	DDT <600 ms on Day 7 after reperfusion was the only indicator of left ventricular remodeling in anterior AMI
Iwakura et al. (51)	2004	DDT of 185 ms	Decreased systolic peak velocity, higher diastolic velocity, shorter DDT, and increased SFR were found more frequently in no-reflow. A DDT <185 ms had good sensitivity and specificity
Hirata et al. (61)	2004	Retrograde diastolic flow in the LAD artery and septal branches	Retrograde flow identified an occluded LAD artery
Nohtomi et al. (59)	2003	Persistent SFR at 48 h	Irreversible dysfunction was predicted more specifically with a persistent SFR at 48 h
Hozumi et al. (50)	2003	DDT cutoff value of 600 ms	After successful coronary angioplasty, the ability of the DDT on Day 1 and Day 3 to identify a viable myocardium had a sensitivity of 78% and 78%, respectively, as well as a specificity of 92% and 84%, respectively
Watanabe et al. (60)	2001	Retrograde flow in the LAD artery	Retrograde flow in the LAD artery was used to diagnose total LAD arterial occlusion
Shintani et al. (55)	2001	Diastolic deceleration half-time of 300 ms	Short diastolic deceleration half-time indicated a poorer functional prognosis after anterior AMI

DDT, diastolic deceleration time; SFR, systolic flow reversal; MACEs, major adverse cardiac events; LAD, left anterior descending; ^|^PDA, posterior descending artery; RCA, right coronary artery; AMI, acute myocardial infarction; CAD, coronary artery disease.

The chronic phase of AMI, which occurs 1 month after the occurrence of AMI, similarly exhibits an abnormal DDT. Giga et al. (53) concluded that the DDT of the LAD artery (infarct-related artery) is associated with the size of the anterior infarction area and the severity and extent of microvascular damage in the chronic duration. A DDT of <886 ms predicts large perfusion abnormalities (defined by an infarct size of >20%) and significant left ventricular contractile impairment (defined by a wall motion score index of >1.5).

DDTs of both the LAD artery and intramyocardial artery may predict wall motion recovery in the infarct area 6 months after anterior AMI. Intramyocardial artery flow, specifically referring to perforator branches, reflects the local myocardial viability, whereas the LAD arterial flow pattern reveals the global myocardial damage (46, 50). An elevated left ventricular end-diastolic pressure may reflect abnormal flow of the LAD and intramyocardial arteries. Severe microvascular damage results in a decreased blood pool, which is rapidly replenished during perfusion. Consequently, the coronary pressure abruptly increases and sharply decreases during the diastolic phase.

Early SFR, as detected by TTE and Doppler guidewire, is one of the powerful predictors of chronic cardiac function and incidence of MACEs in patients with anterior AMI. Previous animal studies have suggested that early SFR indicates myocardial necrosis (45). The presence of early SFR indicated increased microvascular resistance and inadequate perfusion (45, 56). Systolic flow may become fully reversed, extending beyond the early phase as the disease progresses (20). After reperfusion, the microvascular perfusion changes dynamically. Nohtomi et al. (59) reported that the persistence of SFR at Day 2 in reperfused anterior AMI predicts unrecovered myocardial dysfunction more effectively than peak creatine kinase levels, DDT, and immediate SFR. Myocardium damage affects microcirculation resistance, which sharply increases during systolic compression, causing the myocardial blood pool to be squeezed into epicardial coronary arteries instead of coronary veins, ultimately inducing SFR. Compared with a short DDT, SFR causes a more severe obstruction of capacitance vessels.

Unlike AMI, patients with chronic coronary occlusion may not exhibit clinical symptoms, electrocardiogram abnormalities, or regional wall motion abnormalities. Thus, coronary flow detection offers an important clinical tool, owing to its minimal time requirements and low cost. Retrograde coronary flow in both epicardial and intramyocardial arteries has been identified as a meaningful ultrasound index (28, 60, 61). Studies have demonstrated that retrograde blood flow in the epicardial and intramyocardial coronaries has high sensitivity and specificity for diagnosing chronic total coronary occlusion, except in the LCX due to a low success rate of visualization (12).

Takemoto et al. (62) suggested that the AT of systolic coronary flow velocity is a promising marker for diagnosing coronary stenosis accompanied by microvascular dysfunction. Systolic coronary flow is affected by the following three major factors: epicardial artery resistance, intramyocardial artery resistance, and extravascular compression. Previous studies have reported that epicardial coronary flow is influenced by intramyocardial resistance. With regard to extravascular compression, Takemoto et al. reported that the percentage of AT (%AT) in the systolic phase is comparable between patients with reduced LVEF and those with preserved LVEF. Compared with CFVR, the %AT of the systolic phase exhibits similar or even superior diagnostic performance in identifying coronary stenosis (sensitivity,71.1% vs. 83.4%; specificity, 77.3% vs. 71.8%; accuracy, 75.4% vs. 75.4%). Owing to better feasibility of tracing, Takemoto et al. established a cutoff value of 60% for %AT during hyperemia rather than during rest; however, they reported that %AT does not significantly differ between hyperemia and rest. Alterations of CFVR may be related to possible epicardial coronary stenosis and coronary microcirculation abnormality. However, the %AT provides a reliable diagnosis specifically for coronary stenosis accompanied by microvascular dysfunction.

### Cardiomyopathy

Cardiomyopathy is often characterized by myocardial structural and circulatory abnormalities, including fibrosis and restrictive physiology. In addition, individuals with HCM also present with myocardial ischemia, driven by various factors, such as decreased capillary beds, microvascular occlusion, restricted ventricular compliance, and high blood flow demand. Details for resting coronary flow patterns are provided in Table 4.

**Table 4:** Articles on coronary flow patterns in relation to cardiomyopathy in chronological order.

Reference	Year	Results
Galazka et al. (69)	2024	Different flow morphs detected in the septal artery were associated with diastolic dysfunction severity
Peters et al. (67)	2023	A flow pattern with an atrial dip wave in the LAD artery was identified in individuals with HCM
Desai et al. (68)	2017	A flow pattern resembling a square root sign was identified in the septal coronary of individuals with HCM
Aburawi et al. (70)	2009	Increased overall diastolic flow but early termination in endomyocardial fibrosis indicated inadequate myocardium perfusion and further subsequent fibrosis
Kim et al. (65)	2008	A DDT less than 300 ms in the intramyocardial coronary artery and LAD artery indicated steep deceleration and was associated with a higher incidence of symptoms and myocardial ischemia in HCM
Watanabe et al. (66)	2003	HCM was characterized by reverse systolic flow, slow acceleration, and a short DDT in the LAD artery and intramyocardial septal arteries
Crowley et al. (8)	1997	Individuals with HCM presented abnormal flow, including a reduced or reverse systolic peak velocity and prolonged diastolic acceleration and deceleration times
Fusejima (4)	1987	An increased diastolic velocity, an increased velocity–time integrity, and a rectangular flow morph were detected in individuals with HCM

LAD, left anterior descending; HCM, hypertrophic cardiomyopathy; DDT, diastolic deceleration time.

Tomochika et al. (63) observed decreased systolic peak flow velocities or SFR of LAD arteries in individuals with HCM, and they reported a significant inverse correlation between systolic velocity and ventricular septum thickness, as assessed by transesophageal echocardiography. Akasaka et al. (64) corroborated these findings using coronary Doppler catheters. By assessing coronary blood flow using TTE, Crowley et al. (8) reported reversed systolic flow, abnormal velocity, prolonged diastolic AT, and prolonged diastolic deceleration time in HCM.

In HCM, a no-reflow-like pattern in intramyocardial coronaries is highly associated with myocardial ischemia in the absence of epicardial coronary artery stenosis. Two different flow patterns can be observed when focusing on the DDT—a slow deceleration slope and a steep deceleration slope. Kim et al. (65) described a steep slope with a deceleration time of <300 ms in intramyocardial coronaries of HCM patients, indicating a no-reflow-like pattern. They reported that patients with steep deceleration time present more frequent cardiovascular symptoms and a higher incidence of exercise-induced ischemia, as assessed by thallium-201 scintigraphy. Studies have suggested that SFR, diastolic slow acceleration, and rapid deceleration are characteristic changes in intramyocardial coronary and LAD arteries in HCM (32, 66), disagreeing with some previous studies regarding deceleration changes (4, 8). Thus, these findings indicate that there are distinct flow patterns within the same disease on the basis of the extent of disease severity, providing a method for HCM patient stratification.

During reactive hyperemia, accelerated flow velocity persists throughout diastole, resulting in a diastolic flow pattern with a rectangular or mountainous profile. A hallmark rectangular diastolic flow pattern is consistently observed in resting HCM patients, reflecting increased myocardial oxygen demand compared with healthy individuals (4).

In 2023, Tajik et al. (67) identified a distinct and novel flow velocity profile of the LAD artery in HCM—a transient velocity dip in end-diastolic flow accompanied by markedly elevated left ventricular end-diastolic pressure. Coronary blood flow is dependent on coronary perfusion pressure. The transient dip in flow velocity reflects a rapid decrease in coronary perfusion pressure caused by elevated left ventricular end-diastolic pressure secondary to atrial contraction.

The diastolic flow velocities in septal arteries are higher in patients with HCM compared with those in healthy individuals. These velocities in HCM consist of an abrupt reduction after an initial high velocity, followed by a plateau—a pattern that resembles the square root sign (68). Another study has revealed that the detection rate of the septal artery is >50% in HCM and that different diastolic flow patterns of the septal artery are associated with varying grades of diastolic dysfunction (69).

Endomyocardial fibrosis leads to decreased ventricular compliance and increased ventricular diastolic pressure. Aburawi et al. (70) reported increased coronary flow velocity of the LAD artery and PDA, with early termination, in two biventricular affected children, resulting in inadequate perfusion and ultimately fibrosis progression.

### Other diseases

TTE has detected SFR and prolonged DDT in patients with aortic stenosis (71); however, these abnormalities resolve following improvement of stenosis (72). Coronary flow is closely correlated with left ventricular pressure and myocardial ischemia. Systolic coronary flow abnormalities are predictive of prognosis, likely due to microvascular impairment.

Fukuda et al. demonstrated that a hypertrophic heart is characterized by prolonged diastolic AT and late-diastolic step formation, and they reported that these features correlate with the degree of hypertrophy in hypertensive hearts and differ from those in HCM (73). Slow diastolic acceleration indicates a slow pressure gradient between the aorta and the left ventricle, corresponding to a slow decrease in left ventricular pressure. Late-diastolic step formation is detected in hypertension with left ventricular hypertrophy, which is often present in HCM. The late-diastolic step waveform is associated with elevated left ventricular end-diastolic stiffness, indicating elevated left ventricular end-diastolic pressure and decreased perfusion pressure.

Tona et al. (74) first reported that a shorter DDT (<860 ms) predicts future MACEs following heart transplantation. Moreover, cardiac allograft vasculopathy, which affects both epicardial coronary arteries and the microvasculature, is a main limiting factor for long-term survival. Although non-invasive TTE can assess coronary flow abnormalities, further studies are needed to clarify the clinical significance of resting coronary flow patterns in heart transplant recipients.

## Discussion

Recent studies have demonstrated that TTE-derived resting coronary flow parameters (such as velocity, SFR, DDT, and %AT) provide non-invasive insights into coronary hemodynamics and prognostication across various disease states, in particular, CAD and HCM. Coronary flow reflects microvascular function, myocardial ischemia, and perfusion pressure, making it a valuable tool for evaluating cardiovascular diseases and other related disorders.

In CAD, the presence of high-velocity diastolic flow at rest, often accompanied by a mosaic pattern, indicates local coronary stenosis. Further coronary CT angiography and invasive coronary angiography are recommended. Moreover, the resulting increase in the diastolic-to-systolic velocity ratio also suggests the presence of a stenotic lesion. Elevated diastolic flow velocity has also been shown to predict a higher risk of adverse cardiovascular events, potentially due to more severe microvascular impairment. Conversely, extremely low diastolic flow velocity may also signify impaired perfusion, as observed in the slow coronary flow phenomenon. From a spectral morphology perspective, shortened deceleration time in coronary flow indicates more severe microvascular dysfunction and larger areas of infarcted myocardium. Another condition in which resting coronary flow assessment has been applied is HCM. In HCM, a shorter deceleration time reflects poorer myocardial viability, while the appearance of atrial dip and a steep early diastolic drop in the septal coronary flow spectrum are both associated with impaired diastolic function. Assessment of resting coronary flow provides a general understanding of the patient's prognosis, which is helpful for guiding clinical medication and decision-making. In patients with poor reperfusion recovery after the procedure, it also offers a mechanistic explanation. The use of high-resolution ultrasound systems with optimized Nyquist limits and beam alignment protocols enhances the reliability of flow measurements, enabling more precise characterization of microvascular and macrovascular abnormalities. However, several limitations persist. Operator dependency, patient body habitus, and interobserver variability remain significant barriers. This coronary flow imaging technique is highly dependent on the operator's experience, requiring systematic learning and training over a certain period to reliably and efficiently identify the target coronary arteries. In addition, patient-related factors may impose technical limitations: poor acoustic windows or tortuous vessel courses can hinder acquisition of high-quality coronary color Doppler signals. While these parameters show promise, especially in diagnosing and prognosticating CAD, their clinical utility is currently limited due to inconsistent application and low feasibility, likely stemming from a lack of standardized protocols and insufficient awareness among clinicians.

**Figure 4:**
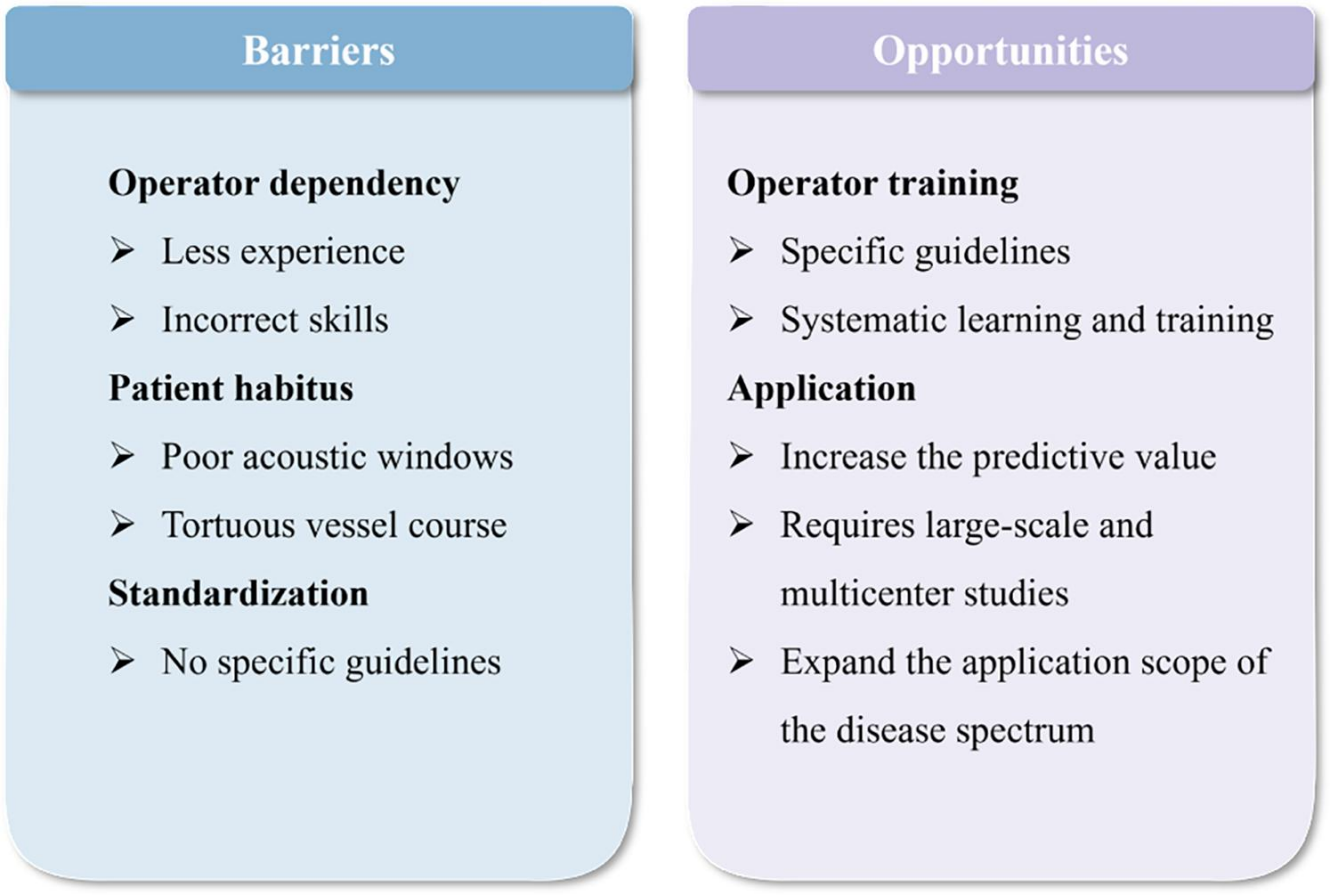
A succinct outline of the barriers and opportunities associated with the application of resting coronary flow.

Most recent studies on resting coronary flow using TTE are mainly observational, single-center studies with small sample sizes, limiting generalizability. The translational gap between technical advancements and clinical practice highlights key areas for future research. Future efforts should focus on translating these technical refinements into standardized clinical workflows to improve the accessibility, reproducibility, and value of resting coronary flow. It is also valuable to elucidate the applications and value of resting coronary flow across a broad spectrum of diseases. Moreover, large-scale and multicenter studies are essential to provide robust evidence for its diagnostic accuracy and prognostic value in predicting adverse cardiovascular events. Figure 4 provides a concise summary of the related barriers and opportunities. As a non-invasive, convenient, and clinically meaningful echocardiographic parameter, resting coronary flow holds great promise and should not be overlooked. Assessment of resting coronary flow characteristics allows evaluation of epicardial coronary stenosis, reflects microvascular function and myocardial perfusion status, and provides prognostic insights into MACEs.
